# Protective Cellular Immune Response Induction for Cutaneous Leishmaniasis by a New Immunochemotherapy Schedule

**DOI:** 10.3389/fimmu.2020.00345

**Published:** 2020-03-03

**Authors:** Danielle A. M. da Silva, Fabiana R. Santana, Simone Katz, Daniel M. Garcia, Daniela Teixeira, Ieda M. Longo-Maugéri, Clara L. Barbiéri

**Affiliations:** ^1^Departamento de Microbiologia, Imunologia e Parasitologia, Escola Paulista de Medicina, Universidade Federal de São Paulo, São Paulo, Brazil; ^2^Departamento de Farmacologia, Escola Paulista de Medicina, Universidade Federal de São Paulo, São Paulo, Brazil

**Keywords:** *Leishmania (Leishmania) amazonensis*, immunochemotherapy, palladacycle complex, rLdccys1, adjuvant *Propionibacterium acnes*

## Abstract

The palladacycle complex DPPE 1.2 was previously shown to inhibit *Leishmania (Leishmania) amazonensis* infection *in vitro* and *in vivo*. The present study aimed to evaluate the effect of DPPE 1.2 associated with a recombinant cysteine proteinase, rLdccys1, and the adjuvant *Propionibacterium acnes* on *L. (L.) amazonensis* infection in two mouse strains, BALB/c, and C57BL/6. Treatment with this association potentiated the leishmanicidal effect of DPPE 1.2 resulting in a reduction of parasite load in both strains of mice which was higher compared to that found in groups treated with either DPPE 1.2 alone or associated with *P. acnes* or rLdccys1. The reduction of parasite load in both mice strains was followed by immunomodulation mediated by an increase of memory CD4^+^ and CD8^+^ T lymphocytes, IFN-γ levels and reduction of active TGF-β in treated animals. No infection relapse was observed 1 month after the end of treatment in mice which received DPPE 1.2 associated with rLdccys1 or rLdccys1 plus *P. acnes* in comparison to that exhibited by animals treated with DPPE 1.2 alone. Evaluation of serum levels of AST, ALT, urea, and creatinine showed no alterations among treated groups, indicating that this treatment schedule did not induce hepato or nephrotoxicity. These data indicate the potential use of this association as a therapeutic alternative for cutaneous leishmaniasis caused by *L. (L) amazonensis*.

## Introduction

Leishmaniasis comprise a group of vector-borne diseases caused by protozoans of *Leishmania* genus and represent an important public health problem with a broad clinical spectrum and epidemiological diversity. The World Health Organization reports a worldwide annual incidence of 0.6–1.0 million of new cases of cutaneous leishmaniasis and 50,000–90,000 of visceral leishmaniasis with the occurrence of 20,000–30,000 deaths (1). Leishmaniasis are included in programs for the control and eradication of neglected tropical diseases.

Among several *Leishmania* species affecting humans, *L. (L.) amazonensis* is one of the causative agents of human cutaneous leishmaniasis in the Amazon region, Brazil, responsible for the development of nodular skin lesions that may evolved to diffuse form (2). Pentavalent antimonials are the first-line drug for the treatment of leishmaniasis, but the high toxicity and low efficacy in immunosuppressed patients have limited their use (3). Amphotericin B and Pentamidine are used to replace antimonials, however, problems such as high cost and toxicity have been reported (4). Miltefosine, initially developed as an antitumor drug, was effective for the treatment of cutaneous and visceral leishmaniasis, representing a promising candidate due to its oral administration, but its use is also limited by problems such as teratogenicity and parasite resistance (5–7). Therefore, it is imperative the development of new therapeutic alternatives for control of leishmaniasis and among them, the development of natural and synthetic products, molecular modifications of existing compounds and drug repositioning have shown different degrees of efficacy in the treatment of experimental leishmaniasis. Antitumor compounds have also shown activity against parasites, including palladacycle complexes that displayed trypanocidal and leishmanicidal effects (8–11). Recent findings identified immunomodulators able to increase the efficacy of leishmanicidal compounds (12, 13).

The activity of two palladacycle complexes, DPPE 1.1 and DPPE 1.2, was previously demonstrated on *in vitro* and *in vivo L. (L.) amazonensis* infection (14, 15). The leishmanicidal effect of these palladacycle complexes is followed by activation of immune responses mediated by an increase of CD4 ^+^ and CD8 ^+^T lymphocytes in treated animals (15, 16). A significant increase in the leishmanicidal effect of DPPE 1.2 as well as of inflammatory responses was observed when this palladacycle complex was associated with heat-killed-*P. acnes* suspension for treatment of *L. (L.) amazonensis*-infected BALB/c and C57BL/6 mice (16). The adjuvant effect of *P. acnes* suspension is due to its ability to increase proinflammatory cytokine synthesis and modulation of Th2 to Th1 responses (17–20). DPPE 1.2 was also used in association with a recombinant cysteine proteinase from *L. (L.) infantum chagasi*, rLdccys1, for treatment of *L. (L.) amazonensis*-infected BALB/c mice. This recombinant protein cross-reacts with a cysteine proteinase of 30 kDa from *L. (L.) amazonesnis* amastigotes and treatment with DPPE 1.2 plus rLdccys1 resulted in a higher leishmanicidal and immunomodulatory effect of this palladacycle complex (21). This recombinant protein is implicated in Th1 lymphocyte-mediated immune responses in patients and dogs with visceral leishmaniasis (22), as well as in protective responses in mice and dogs previously immunized and challenged with *L. (L.) infantum chagasi* (23, 24). The efficacy of the treatment of canine visceral leishmaniasis with this recombinant protein plus *P. acnes* was also demonstrated (25). These findings led us to use DPPE 1.2 associated with rLdccys1 and *P. acnes* for the treatment of *L. (L.) amazonensis*-infected mice. This treatment schedule resulted in the exacerbation of the leishmanicidal effect of DPPE 1.2 followed by an increase of memory CD4^+^ and CD8^+^ T lymphocytes, IFN-γ levels and reduction of active TGF-β in treated animals.

## Materials and Methods

### Experimental Animals

Female BALB/c and C57BL/6 mice 6 to 8 weeks old were acquired from Universidade Federal de São Paulo (São Paulo, Brazil). Eight-week-old female Golden hamsters were obtained from Anilab Company, Paulínia (São Paulo, Brazil). All animals were maintained under specific pathogen-free conditions and fed a balanced diet. All animal procedures were carried out in accordance with the recommendations in the Guide for the Care and Use of Laboratory Animals of the Brazilian National Council of Animal Experimentation. The protocol was approved by the Committee on the Ethics of Animal Experiments of the Institutional Animal Care and Use Committee at the Federal University of São Paulo (Id # CEUA: 2957181016).

### Parasites

The *L. (L.) amazonensis* strain used (MHOM/BR/1973/M2269) was kindly provided by Dr. Jeffrey Jon Shaw, Instituto Evandro Chagas, Belém, Pará, Brazil and maintained as amastigotes by inoculation into footpads of Golden hamsters every 4 to 6 weeks as previously described (26).

### Biphosphinic Palladacycle Complex [Pd (C2, N-S(-)DMPA)(DPPE)]Cl (DPPE 1.2)

The palladacycle compound DPPE 1.2 was obtained from N,N-dimethyl-1-phenylethylamine (DMPA), complexed to 1,2-ethane-bis(diphenylphosphine; DPPE) ligand and synthesized as previously described (27). Stock solutions at 1.45 mM were prepared in phosphate-buffered saline (PBS; 137 mM NaCl in 10 mM phosphate buffer, pH 7.4) after solubilization in dimethylsulfoxide (final concentration of 0.1%).

### Heat-Killed *P. acnes* Suspension

*Propionibacterium acnes* was obtained from Instituto Adolfo Lutz, São Paulo, S.P., Brazil and cultured in anaerobic medium (Hemobac, Probac, São Paulo, S.P., Brazil) for 3 days at 37°C and washed by centrifugation (28). The resulting pellets were suspended in 0.9% saline and subjected to continuous water vapor for 20 min at 120°C. The protein concentration of the suspension was determined by the Bradford method (29).

### Expression and Purification of Recombinant Cysteine Proteinase (rLdccys1)

The PCR product corresponding to the open reading frame (ORF) of the *L. (L.) infantum chagasi* Ldccys1 gene previously obtained in our laboratory (30) was subcloned into the *Bam*HI and *Eco*RI restriction sites of the parallel pHIS 3 expression vector containing a tail of six histidine residues in the amino-terminal region (31). Recombinant plasmids were used to transform *Escherichia coli* BL21 (DE3). Protein expression was performed by inoculating 25 ml of a bacterial culture into 500 ml LB medium containing 100 μg/ml ampicillin. The suspension was kept on a shaker at 37°C until reaching the log phase (absorbance at 600 nm = 0.6) and protein expression was induced with 1 mM IPTG for a further 3 h at 37°C. After growth, the recombinant bacteria was pelleted at 4,000 × *g* for 10 min and the recombinant antigen was purified from insoluble inclusion bodies treated with solubilization buffer (0.1 M NaH_2_PO_4_, 10 mM Tris–HCl, pH 8.0, 8M urea). Purification of the recombinant protein was performed by affinity chromatography using Ni-NTA Superflow Agarose Matrix (QIAGEN) as previously described (32). Purified protein was analyzed by SDS-PAGE and Western blotting using mouse serum anti-rLdccys1. Cross-reactivity of rLdccys1 and a 30 kDa cysteine proteinase from *L. (L.) amazonensis* was demonstrated in amastigote extracts of this parasite subjected to SDS-PAGE and Western blotting (21).

### *In vivo* Antileishmanial Assays

*In vivo* leishmanicidal activity of DPPE 1.2 associated with rLdccys1 plus *P. acnes* was evaluated in female BALB/c and C57BL/6 mice infected subcutaneously at the right hind-foot pad with 1 × 10^5^ *L. (L.) amazonensis* amastigotes freshly isolated from infected Golden hamsters as previously described (14). Twenty days after infection, the animals were separated into eight groups of 10 mice each. Treated animals received in the foot lesions every other day 21 doses of 160 μg/Kg of DPPE 1.2 (total of 3.36 mg/Kg) associated or not with 50 μg/animal of rLdccys1 and 100 μg/animal of *P. acnes*. The recombinant antigen and the adjuvant were administered on the 1st, 7th, and 14th days of treatment (total of 150 and 300 μg/animal, respectively). The infection was monitored once a week by measuring the diameter of foot lesions with a dial caliper (Mitutoyo Corp., Japan). Parasite burden was evaluated by limiting dilution in foot lesions of BALB/c and C57BL/6 mice after the end of the treatment, as previously described (33). Treatment longevity was evaluated in the remaining animals 30 days after the end of treatment. During this period, the groups treated with DPPE 1.2 associated with either the recombinant protein, *P. acnes* or both received once a week additional doses of either 50 μg of rLdccys1, 100 μg of *P. acnes* or 50 μg of rLdccys1 plus 100 μg of *P. acnes*.

### Evaluation of Immune Responses

T lymphocyte populations were evaluated by flow cytometry. Popliteal and inguinal lymph nodes were isolated from BALB/c and C57BL/6 mice. After staining with Trypan Blue and counting in a Neubauer chamber, 1 × 10^6^ lymphocytes were washed twice in PBS and incubated with monoclonal antibodies either anti-CD3 conjugated to allophycocyanin (APC), anti-CD4 conjugated to phycoerythrin cyanine 5 (PECy5), anti-CD8 conjugated to pacific blue (PB), anti-CD44 conjugated to phycoerythrin cyanine 7 (PeCy7) (Pharmingen) or anti-CD62L conjugated to phycoerythrin (PE) (Pharmingen). After incubation for 30 min at 4°C, the cells were washed in PBS and fixed in formalin 1%. Fluorescence intensity was analyzed by FACS (FACSCAN – Cell Sorter Becton–Dickinson). Detection of IFN-γ and active TGF-β was carried out in the supernatants of foot lesions from treated BALB/c and C57BL/6 mice. The animals were euthanized, and after homogenization of excised foot lesions in PBS, supernatants were collected, cleared by centrifugation and assayed for these cytokines using a double-sandwich ELISA assay according to the manufacturer instructions (eBioscience, Inc., San Diego, CA, United States). Supernatant concentrations higher than the minimal values obtained from standard IFN-γ and TGF-β were considered positive.

For evaluation of proliferation in response to rLdccys1, lymphocytes isolated from popliteal and inguinal lymph nodes were stained with 1.5 μM of carboxyfluorescein succinimidyl ester (CFSE) and cultured (5 × 10^5^ cells/well) with 5 μg/ml of rLdccys1 peptides or medium (R10) alone for 3 days under an atmosphere of 5% CO_2_ at 37°C. Cells were then collected and labeled for 30 min at 4°C with anti-CD3 conjugated to allophycocyanin (APC), anti-CD4 conjugated to phycoerythrin cyanine 5 (PECy5), anti-CD8 conjugated to pacific blue (PB), anti-CD44 conjugated to phycoerythrin cyanine 7 (PeCy7) (Pharmingen) and anti-CD62L conjugated to phycoerythrin (PE) (Pharmingen). After washing, samples were analyzed using a FACSCanto^TM^ II (BD) with appropriate compensation controls (single stained beads, CompBeads (BD), or CFSE-single stained cells). The frequency of proliferating T lymphocyte subpopulations (CFSE^low^) among populations was determined using FlowJo software, version 9.7.6 (Tree Star). The rLdccys1 specific population was determined by estimating the number of rLdccys1-stimulated CFSE^low^ cells subtracted from numbers of unstimulated lymphocytes.

### Toxicity Assays

Serum concentrations of urea, creatinine, and transaminases were determined in BALB/c and C57BL/6 mice at the end of treatment, using sets of commercial reagents (Labtest, Ltda, Brazil).

### Statistical Analysis

The data were analyzed using GraphPad Version 8.0.0 software, applying one-way ANOVA and Tukey’s multiple comparison test (*P* < 0.001).

## Results

### Association With rLdccys1 Plus *P. acnes* Increased the Leishmanicidal Effect of DPPE 1.2 in *L. (L.) amazonensis*-Infected BALB/c and C57BL6 Mice

**FIGURE 1:**
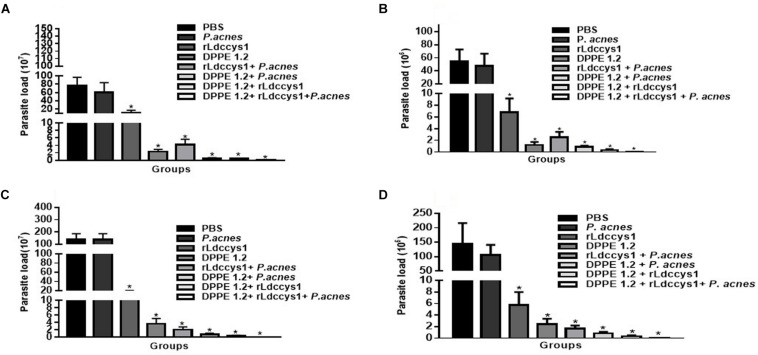
Effect of DPPE 1.2 associated with rLdccys1 plus *P. acnes* on *L. (L.) amazonensis*-infected BALB/c **(A,C)** and C57BL/6 mice **(B,D)**. Parasite load in foot lesions evaluated 3 days (Phase 1) **(A,B)** and 30 days after the end of treatment (Phase 2) **(C,D)**. ^∗^*P* < 0.001. Data are representative of three independent experiments.

The treatment with DPPE 1.2 associated with rLdccys1 plus *P. acnes*, hereafter referred as triple association, was performed by administration of 3.36 mg/Kg of DPPE 1.2, 150 mg/animal of rLdccys1 and 300 mg/animal of *P. acnes*. The parasite load of BALB/c mice evaluated 3 (phase 1) and 30 days after the end of treatment (phase 2) was 14 and 19 times, respectively, higher than that found in C57BL/6 mice. In both mouse strains, there were no significant differences between the parasite load of animals treated with either PBS or *P. acnes*. The other treated groups from both strains evaluated in phase 1 showed a significant reduction of parasite load compared to controls. BALB/c and C57BL/6 mice treated with the triple association displayed a parasite reduction of 1935 and 1265 times, respectively, compared to the parasite load found in controls. Although the parasite load from the group treated with the triple association was lower than that observed in the other treated groups, this difference was not statistically significant (Figure 1). Control groups, as well as those treated either with DPPE 1.2 alone or DPPE 1.2 plus *P.* acnes from both mouse strains, displayed an increase of parasite load in phase 2. On the other hand, all groups treated with either rLdccys1 alone or associated with DPPE 1.2, *P. acnes* or both showed a parasite load very similar to that evaluated in phase 1 (Table 1).

**TABLE 1:** Parasite load in foot lesions of *L. (L.) amazonensis*-infected BALB/c and C57BL/6 mice treated with DPPE 1.2 associated with rLdccys1 plus *P. acnes* evaluated in phases 1 and 2.

Groups	Parasite load BALB/c (1.10^7^) Phase 1	Parasite load BALB/c (1.10^7^) Phase 2	Parasite load C57BL/6 (1.10^6^) Phase 1	Parasite load C57BL/6 (1 10^6^1 Phase 2
PBS	77,4 ± 19,23	136 ± 49,29	54,4 ± 18,62	144,1 ± 71,75
*P. acnes*	60,2 ± 23,55	136 ± 49,29	47,6 ± 18,62	104,8 ± 35,87
rLdccysl	14 ± 5,75	14,7 ± 5,75	5,06 ± 2,35	6,78 ± 2,24
DPPE 1.2	2,34 ± 0,57	3,68 ± 1,45	1,11 ± 0,49	2,95 ± 0,91
rLdccysl + *P. acnes*	4,19 ± 1,45	2,08 ± 0,71	0,34 ± 0,93	0,354 ± 0,55
DPPE *1.2* + *P. acnes*	0,518 ± 0,18	0,78 ± 0,29	2,52 ± 0,21	1,63 ± 0,27
DPPE 1.2 + rLdccysl	0,518 ± 0,18	0,354 ± 0,20	0,81 ± 0,17	0,9 ± 0,14
DPPE 1.2 + rLdccysl + *P. acnes*	0,04 ± 0,024	0,027 ± 0,008	0,043 ± 0,014	0,044 ± 0,017

*Values represent mean ± standard deviation among 5 animals per group.*

**FIGURE 2:**
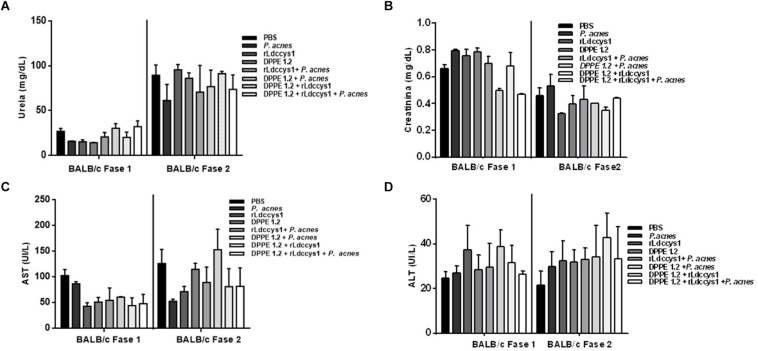
Evaluation of toxicity in *L. (L.) amazonensis*-infected BALB/c mice after treatment with DPPE 1.2 associated with rLdccys1 plus *P. acnes*. Serum concentrations of urea **(A)**, creatinine **(B)**, AST **(C)**, and ALT **(D)** in phases 1 and 2. The reference values are: urea: 42–55 mg/dL; creatinine: 0,44–0,55 mg/dL; AST: 70–100 UI/L; ALT: 30–35 UI/L.

**FIGURE 3:**
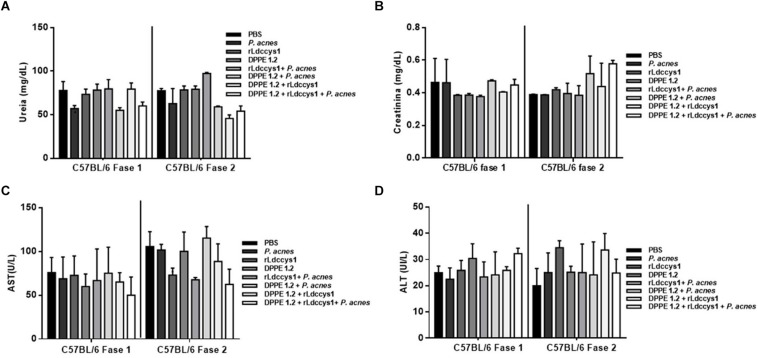
Evaluation of toxicity in *L. (L.) amazonensis*-infected C57BL/6 mice after treatment with DPPE 1.2 associated with rLdccys1 plus *P. acnes*. Serum concentrations of urea **(A)**, creatinine **(B)**, AST **(C)**, and ALT **(D)** in phases 1 and 2. The reference values are: urea: 42–55 mg/dL; creatinine: 0,44–0,55 mg/dL; AST: 70–100 UI/L; ALT: 30–35 UI/L.

Determination of hepato and nephrotoxicity of treated animals by detection of serum levels of transaminases, urea and creatinine showed no statistically significant alterations among controls and treated groups (Figures 2, 3).

### Increase of CD4^+^ CD8^+^ and Memory T lymphocytes in *L. (L.) amazonensis*-Infected BALB/c and C57BL/6 Mice After Treatment With DPPE 1.2 Associated With rLdccys1 Plus *P. acnes*

**FIGURE 4:**
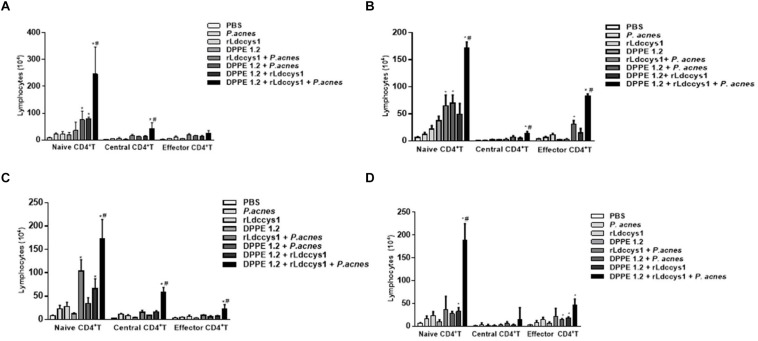
Analysis of central and effector memory CD4^+^ T lymphocytes in *L. (L.) amazonensis*-infected BALB/c **(A,C)** and C57BL/6 **(B,D)** mice treated with DPPE 1.2 associated with rLdccys1 plus *P. acnes* evaluated in phases 1 **(A,B)** and 2 **(C,D)**. ^∗^*P* < 0.001 compared to control; ^#^*P* < 0.001 compared to the other treated groups.

**FIGURE 5:**
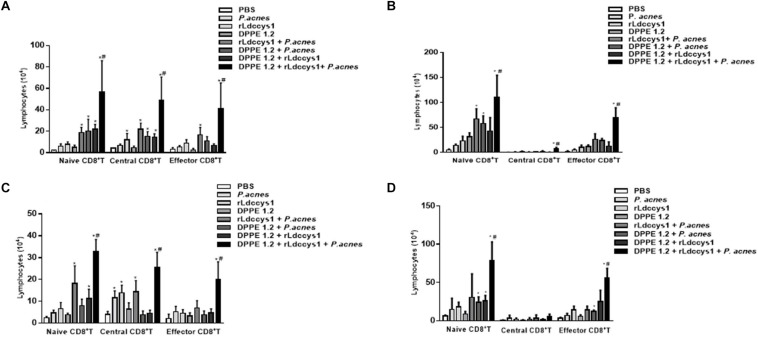
Analysis of central and effector memory CD8^+^ T lymphocytes in *L. (L.) amazonensis*-infected BALB/c **(A,C)** and C57BL 6 **(B,D)** mice treated with DPPE 1.2 associated with rLdccys1 plus *P. acnes* in phases 1 **(A,B)** and 2 **(C,D)**. ^∗^*P* < 0.001 compared to control groups; ^#^*P* < 0.001 compared to the other treated groups.

Data on analysis of CD4^+^ and CD8^+^ T lymphocyte expression are shown in Figures 4, 5, and Supplementary Figures S1–S5, respectively. There was an increase of CD4^+^ T lymphocytes in some groups of treated BALB/c mice compared to controls and this increase was significantly higher in those treated with the triple association in both evaluation phases. Central memory CD4^+^ T lymphocytes increased only in BALB/c mice treated with the triple association in the two evaluation phases, while in this group memory effectors increased significantly only in phase 2 (Figures 4A,C).

Some groups of treated C57BL/6 mice displayed an increase of CD4^+^ T lymphocytes compared to control groups and this increase was significantly higher in those treated with the triple association in both phases of evaluation. There was an increase of central memory CD4^+^ T lymphocytes during phase 1 in the group treated with the triple association, whereas this group displayed a significant increase of effector memory CD4^+^ T lymphocytes in both evaluation phases (Figures 4B,D).

Regarding CD8^+^ T lymphocytes, although there was an increase of this population in some groups of treated BALB/c mice, this increase was significantly higher in those treated with the triple association in both phases of parasite evaluation. The central and effector memory CD8^+^ T lymphocytes also increased only in the group treated with the triple association in both evaluation phases (Figures 5A,C).

In C57BL/6 strain there was a significant increase of CD8^+^ T population in some of the treated groups which was significantly higher in those treated with the triple association. A significant increase of central memory CD8^+^ T lymphocytes was observed in the group treated with the triple association during phase 1, whereas effector memory CD8^+^ T lymphocytes increased in both phases of evaluation (Figures 5B,D). It was also demonstrated that in phase 2 the C57BL/6 strain displayed a higher number of effector memory CD8^+^ T lymphocytes compared to that found in BALB/c mice (Figures 5C,D).

### Lymphoproliferative Responses to rLdccys1 From *L. (L.) amazonensis*-Infected BALB/c Mice Treated With DPPE 1.2 Associated With rLdccys1 Plus *P. acnes* Resulted in an Increase of CD4^+^, CD8^+^, and Memory T Lymphocytes

**FIGURE 6:**
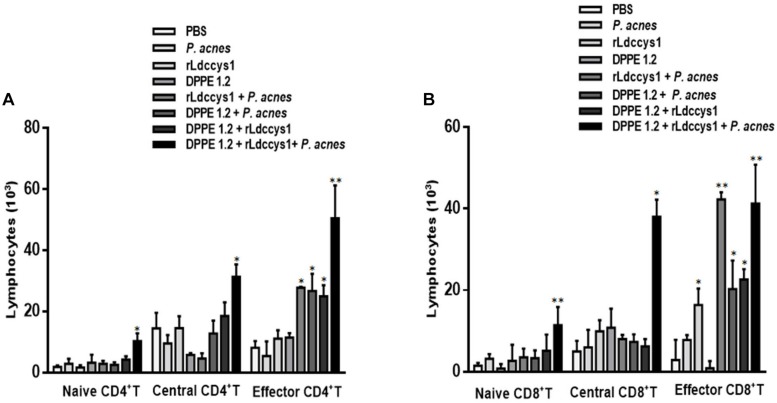
Proliferative responses to rLdccys1 of lymphocytes isolated from inguinal and popliteal lymph nodes of *L. (L.) amazonensis*-infected BALB/c mice 1 month after the end of treatment with DPPE 1.2 associated with rLdccys1 plus *P. acnes*. Data are representative of two experiments and were defined by CFSE dilution in gated CD3^+^CD4^+^ and CD3^+^CD8^+^ cells by flow cytometry. Memory T population was defined in gated CD44^low^ CD62L^high^ for naive, CD44^high^ CD62L^high^ for central and CD44^high^ CD62L^low^for effectors. Results are presented as values (mean ± SD) for each group. ^∗^*P* < 0.001.

Proliferative responses were evaluated in lymphocytes isolated from inguinal and popliteal lymph nodes of *L. (L.) amazonensis-infected* BALB/c mice 30 days after treatment with DPPE 1.2 associated with rLdccys1 plus *P. acnes* by stimulation *in vitro* with rLdccys1. These data are shown in Figure 6 and Supplementary Figures S6, S7. Cells from animal group treated with the triple association measured 3 days after rLdccys1 stimulation resulted in a significant increase of CD4^+^ T and central memory CD4^+^ T lymphocytes while increase of effector memory CD4^+^ T was observed in cell cultures from groups treated with rLdccys1 plus either *P. acnes* or DPPE 1.2, besides from that treated with DPPE 1.2 plus *P. acnes*, but this increase was significantly higher in lymphocytes from mice treated with the triple association (Figure 6A).

The proliferative responses under rLdccys1 stimulation increased CD8^+^ T and central CD8^+^ T lymphocytes from the group treated with the triple association. Although there was also proliferation of effector CD8^+^ T lymphocytes stimulated with rLdccys1 in groups treated with either rLdccys1 alone or associated with DPPE 1.2, as well as with DPPE 1.2 plus *P. acnes*, these lymphoproliferative responses were significantly higher in groups treated with either rLdccys1 plus *P. acnes* or the triple association (Figure 6B).

### Treatment of *L. (L.) amazonensis*-Infected BALB/c and C57BL/6 Mice With DPPE 1.2 Associated With rLdccys1 Plus *P. acnes* Resulted in an Increase of IFN-γ and Reduction of TGF-β Secretion

**FIGURE 7:**
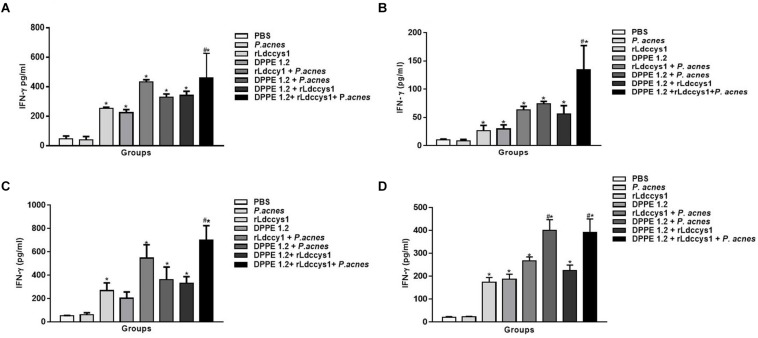
Secretion of IFN-γ in treated mice. Levels of IFN-γ were determined by ELISA assay in supernatants of foot lesions from *L. (L.) amazonensis*-infected BALB/c **(A,C)** and C57BL/6 **(B,D)** mice treated with DPPE 1.2 associated with rLdccys1 plus *P. acnes* in phases 1 **(A,B)** and 2 **(C,D)**. ^∗^*P* < 0.001 compared to control groups; ^#^*P* < 0.001 compared to the other treated groups.

Treated groups from BALB/c and C57BL/6 mice, in both evaluation phases, displayed high levels of IFN-γ in foot lesions compared to controls and in mice treated with the triple association IFN-γ secretion was significantly higher compared to that secreted by the other treated groups (Figure 7). It was also observed that C57BL/6 mice treated with DPPE 1.2 plus *P. acnes* exhibited levels of IFN-γ comparable to those found in the group treated with the triple association (Figure 7D).

**FIGURE 8:**
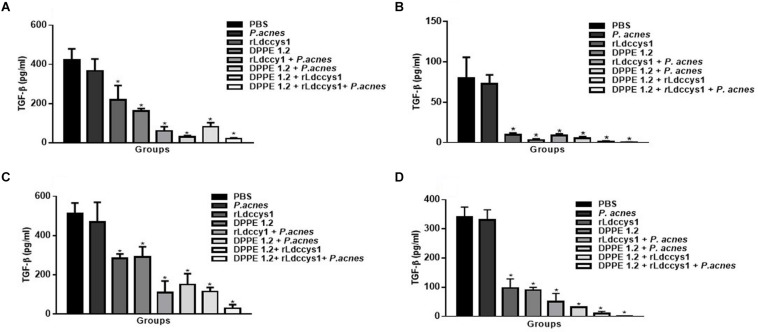
Evaluation of active TGF-β in treated mice. Levels of active TGF-β were determined by ELISA assay in supernatants of foot lesions from *L. (L.) amazonensis*-infected BALB/c **(A,C)** and C57BL/6 mice **(B,D)** treated with DPPE 1.2 associated with rLdccys1 plus *P. acnes* in phases 1 **(A,B)** and 2 **(C,D)**. ^∗^*P* < 0.001 compared to control groups.

As can be seen in Figure 8 there was a significant decrease of TGF-β secretion in treated animals from both mouse strains in the two evaluation phases compared to that detected in controls. Although the reduction of TGF-β was more pronounced in groups treated with the tripled association in comparison to the other treated groups, this difference was not statistically significant. It was also noted that in both evaluation phases TGF-β levels were higher in all groups of BALB/c mice compared to those found in C57BL/6 mice.

## Discussion

In the present study, the potentiation of the leishmanicidal effect of DPPE 1.2 was demonstrated in *L. (L.) amazonensis*-infected BALB/c and C57BL/6 mice after treatment with this palladacycle complex associated with rLdccys1 plus *P. acnes*. The reduction of parasite load in treated groups was followed by immunomodulation evidenced in these animals by the significant increase of CD4^+^ and CD8^+^ T lymphocytes and IFN-γ levels and a concomitant reduction of active TGF-β. Increase of CD4 ^+^ T lymphocytes, as well as IFN-γ production in groups treated with *P. acnes* associated with either DPPE 1.2, rLdccys1 or both corroborate previous data showing that the treatment with this adjuvant induces immune responses mediated by Th1 lymphocytes with IFN-γ production (17). In experimental leishmaniasis, *P. acnes* was used as an adjuvant with native and recombinant *Leishmania* antigens for mice vaccination that resulted in protective immunity mediated by CD4^+^ Th1 lymphocytes (23, 34). More recently, the potentiation of the leishmanicidal effect of DPPE 1.2, as well as immune responses mediated by CD4^+^ T lymphocytes with increased IFN-γ production, was demonstrated in mice treated with *P. acnes* associated with DPPE 1.2 (16). The treatment with rLdccys1 alone or associated with *P. acnes*, DPPE 1.2, or both led to IFN-γ production, corroborating our previous findings which demonstrated a predominance of inflammatory responses in mice immunized with rLdccys1, as well as in *L. (L.) infantum chagasi*-infected dogs treated with this recombinant antigen (23, 25). Furthermore, although not statistically significant, the treatment with rLdccys1 induced an increase of CD8^+^ T lymphocytes and this increase was significantly higher in mice treated with either rLdccys1 plus *P. acnes* or the triple association. These data are in accordance with previous findings on analysis of the predicted amino acid sequence of the cloned *L. (L.) infantum chagasi Ldccys1* gene which showed one potential MHC class I epitope for CD8^+^ T lymphocytes besides two MHC class II epitopes (30). In addition, parallel to its inflammatory property, *P. acnes* increases MHCI, MHCII, TLR2.4.9 molecules expression in cell surface as dendritic cells macrophages and B lymphocytes (20, 35, 36). The IFN-γ production suggests its involvement in the destruction of parasites in treated animals. Although the participation of CD4^+^ Th1 lymphocytes with IFN-γ production has been demonstrated in mice protected against *L. (L.) amazonensis* infection (37, 38), it is important to emphasize that the immune responses induced against *L.* (*L.*) *amazonensis* have shown a distinct pattern from that described for *L.* (*L.*) *major*. While mice that are resistant or susceptible to infection with *L.* (*L.*) *major* exhibit immune responses polarized to Th1 and Th2, respectively, susceptibility to *L.* (*L.*) *amazonensis* cannot be correlated to an increased Th2 response (39). Furthermore, BALB/c mice infected with *L.* (*L.*) *amazonensis* develop Th1 responses (39–41) and the treatment with either IFN-γ or IL-12 did not affect the outcome of *L.* (*L.*) *amazonensis* infection in BALB/c and C57BL/6 mice (42, 43). These data and recent findings that showed the resistance of the *L. (L.) amazonensis* strain used in the present study to the action of nitric oxide secreted by activated macrophages (44) permit to ascribe to cytotoxic CD8 ^+^ T lymphocytes a more relevant role in reduction of parasitic load. Other literature data have also shown that the susceptibility of *L. (L.) amazonensis* to nitric oxide secreted by activated macrophages is controversial (45, 46). The hypothesis of the relevance of cytotoxic CD8 ^+^ T lymphocytes in the destruction of parasites in treated animals is corroborated by previous data showing an increase of this lymphocyte population in parallel with its cytotoxic activity on *L. (L.) amazonensis* infected macrophages from partially protected BALB/c mice against homologous infection (47). Furthermore, the relevant role of cytotoxic CD8^+^ T lymphocytes producing perforin and IFN-γ on *L. (L.) amazonensis* infection has been demonstrated by other authors (48–50).

The specificity to rLdccys1 of the immune responses observed in treated groups was demonstrated by the evaluation of proliferative responses of lymphocytes from *L. (L.) amazonensis*-infected mice 30 days after the end of treatment stimulated *in vitro* by the recombinant antigen. Animal group treated with the triple association showed a significant lymphoproliferative responses mediated by CD4^+^, central CD4^+^ memory T cells, while all groups that received the recombinant protein displayed proliferative responses mediated by effector CD4^+^ memory T lymphocytes. These results are probably related to the permanent presence of the antigen *in vivo* during the treatment. The group treated with DPPE 1.2 plus *P. acnes* also exhibited effector CD4^+^ memory T responses possibly due to the immunological properties of this adjuvant previously discussed. Similar proliferative responses mediated by CD8^+^ T lymphocytes stimulated by rLdccys1 were also observed.

The implication of TGF-β in increasing infection by cutaneous and visceralizing *Leishmania* species has been demonstrated (51, 52). Data from the present study corroborate these results, as low levels of TGF-β were detected in lesions of treated mice that presented reduced parasitic load, a concomitant increase of CD4^+^T and CD8^+^T lymphocytes and a significant IFN-γ secretion. BALB/c mice displayed about 10 times higher parasite load compared to C57BL/6. These results reflect the different susceptibility to *L. (L.) amazonensis* of these strains since BALB/c mice develop the diffuse anergic form of cutaneous leishmaniasis characterized by metastatic and non-curable lesions with the presence of a high number of amastigotes (53). On the other hand, infection with *L. (L.) amazonensis* in the C57BL/6 strain leads to the development of chronic lesions (54). IFN-γ secretion was higher in BALB/c mice compared to C57BL/6, but in both strains there was a significant increase of this cytokine in animals treated with the triple association. On the other hand, in phase 1 of evaluation the TGF-β levels of C57BL/6 mice were about 5 times lower compared to those exhibited by BALB/c mice, possibly indicating that a lower immunosuppressive environment leads to the higher resistance of the C57BL/6 strain to *L. (L.) amazonensis* infection.

Regarding the treatment longevity, the data showed that all animals treated with DPPE 1.2 which received rLdccys1, *P. acnes*, or both for more 30 days displayed a parasite load similar to that observed in the first phase of evaluation, whereas there was a relapse in those animals treated with DPPE 1.2 alone. The increase of memory CD4^+^ T lymphocytes was only observed in animals treated with the triple association and among them, effector memory CD4^+^ T lymphocytes were detected only in C57BL/6 mice in both phases, whereas in BALB/c mice this set of lymphocytes increased only in phase 2 of evaluation. In the two mouse strains treated with the triple association, there was an increase of central and effector memory CD8^+^ T lymphocytes in both evaluation phases. In BALB/c mice the number of central memory CD8^+^ T lymphocytes was similar in both evaluation phases, whereas in C57BL/6 mice there was a higher proportion of effector memory CD8^+^ T lymphocytes in phase 1 of evaluation and in phase 2 only the effectors persisted. These findings are in accordance with the significant secretion of IFN-γ and low levels of TGF-β in mice treated with the triple association, indicating the maintenance of an inflammatory response in these groups which was more quickly induced in the C57BL/6 strain. The involvement of memory lymphocytes in the elimination of microorganisms is closely related to the prompt and specific nature of their responses and they are generated according to the intensity and maintenance of the inflammatory stimulus. Thus, the association of DPPE 1.2 with rLdccys1 plus *P. acnes* was able to potentiate and keep this stimulus due to the pro-inflammatory nature of this adjuvant and rLdccys1. Recently we also detected specific memory and effector CD4^+^ and CD8^+^ T lymphocytes increased in a HIV vaccine associated with *P. acnes* in experimental model including immune response longevity (55). In animals that received only DPPE 1.2, the inflammatory environment was not sustained, allowing parasite replication and disease relapse.

The present data are supported by literature reports which have shown the advantage of immunochemotherapy over monotherapy both in murine models of cutaneous and visceral leishmaniasis, as well as in patients suffering from American cutaneous leishmaniasis treated with leishmanicidal drugs associated with different *Leishmania* antigens (56–61).

## Conclusion

In conclusion, data from the present study strengthened the effective inhibitory action of DPPE 1.2 on *in vivo L. (L.) amazonensis* infection at non-toxic concentrations. The association of this palladacycle complex with rLdccys1 plus *P. acnes* potentiated the leishmanicidal effect of DPPE 1.2, opening perspectives to further explore this association as a therapeutic alternative for cutaneous leishmaniasis caused by *L. (L.) amazonensis*.

## Data Availability Statement

All datasets generated for this study are included in the article/Supplementary Material.

## Ethics Statement

This study was carried out in strict accordance with the National Institutes of Health Guide for the Care Use of Laboratory Animals and the Brazilian National Law (11.794/2008). The protocol was approved by the Committee on the Ethics of Animal Experiments of the Institutional Animal Care and Use Committee at the Federal University of São Paulo (Id # CEUA: 2957181016).

## Author Contributions

DS, FS, DT, and SK designed and performed the experiments, and analyzed and interpreted the data. DG designed, synthesized, and analyzed the compounds. IL-M analyzed and interpreted the data, and contributed to reviewing of the manuscript. CB and DG conceived the work, contributed to interpretation of the data, and wrote and reviewed the manuscript.

## Conflict of Interest

The authors declare that the research was conducted in the absence of any commercial or financial relationships that could be construed as a potential conflict of interest.
