# Supplementary figures and images for: Protective Cellular Immune Response Induction for Cutaneous Leishmaniasis by a New Immunochemotherapy Schedule
Source: Front Immunol. 2020 Mar 3;11:345. doi: 10.3389/fimmu.2020.00345 (PMC7062680; doi:10.3389/fimmu.2020.00345)

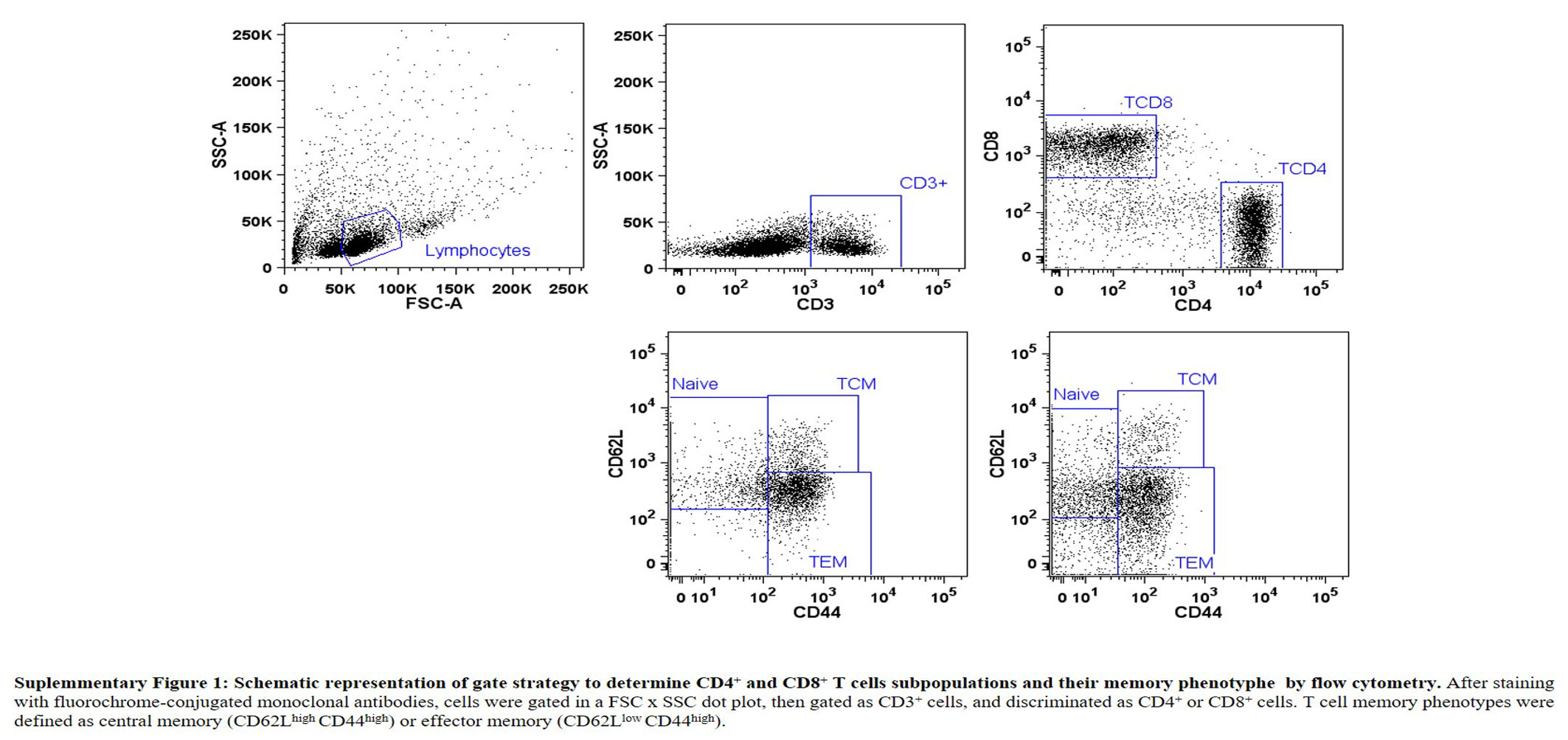

Supplement: Supplementary file 1 [file Image_1.JPEG]

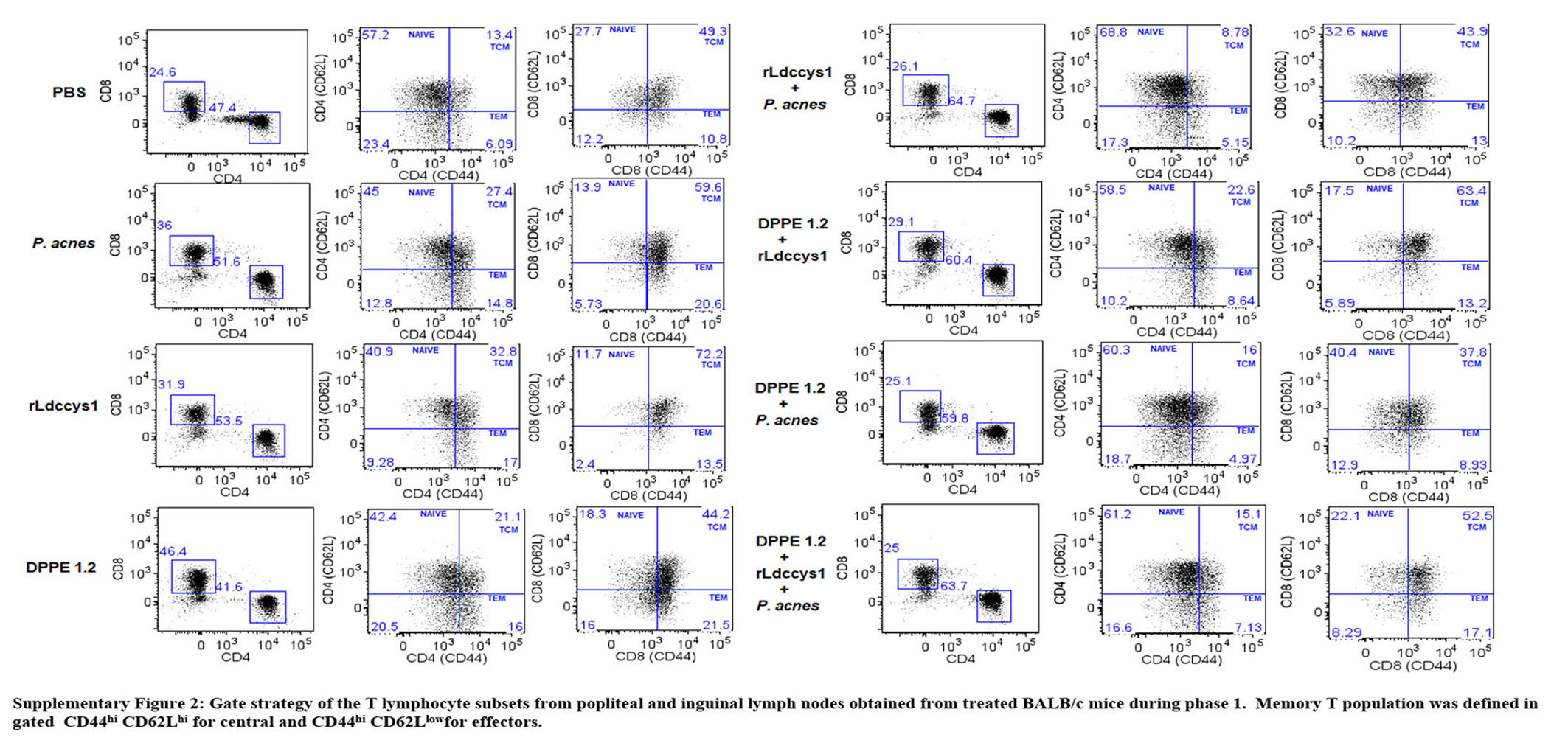

Supplement: Supplementary file 2 [file Image_2.JPEG]

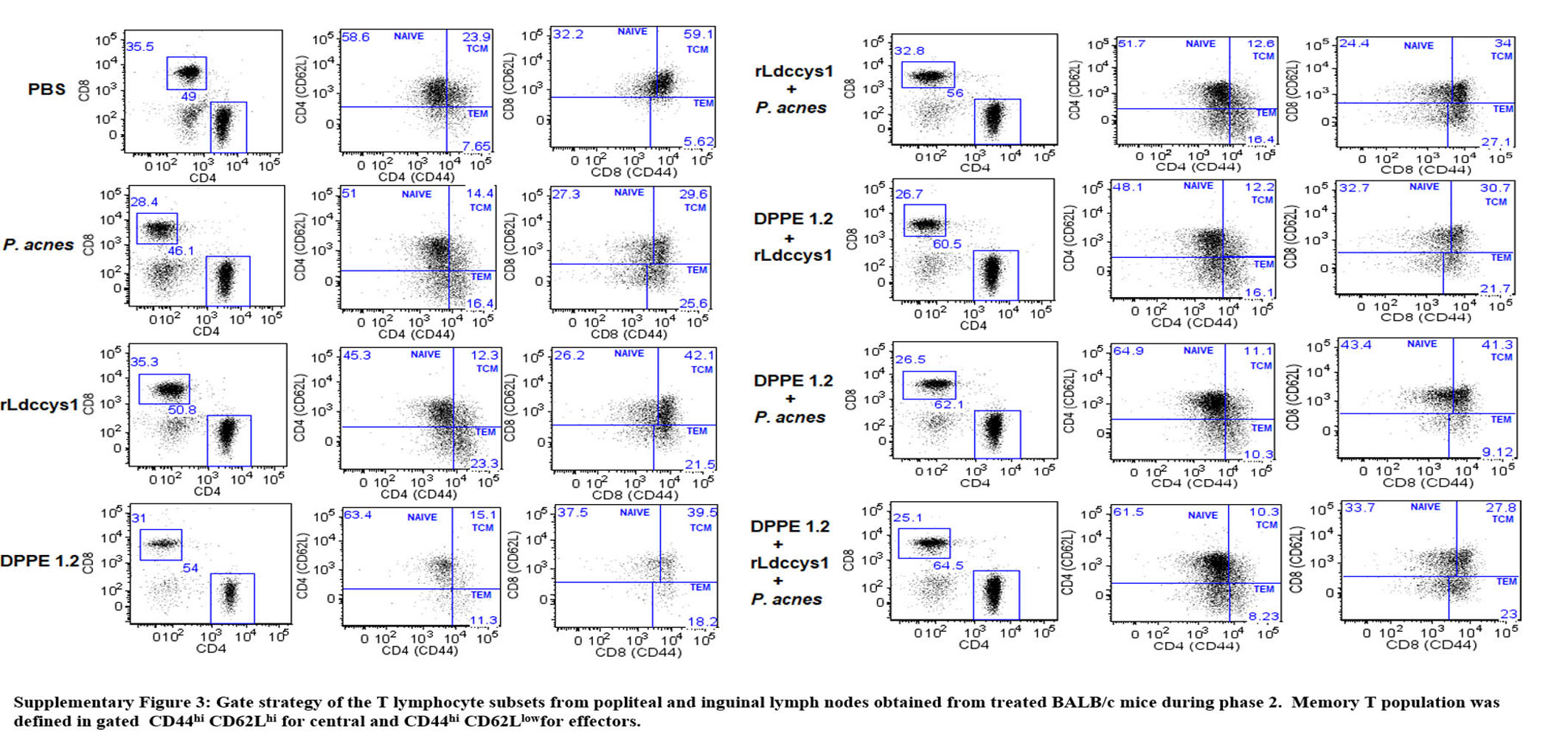

Supplement: Supplementary file 3 [file Image_3.JPEG]

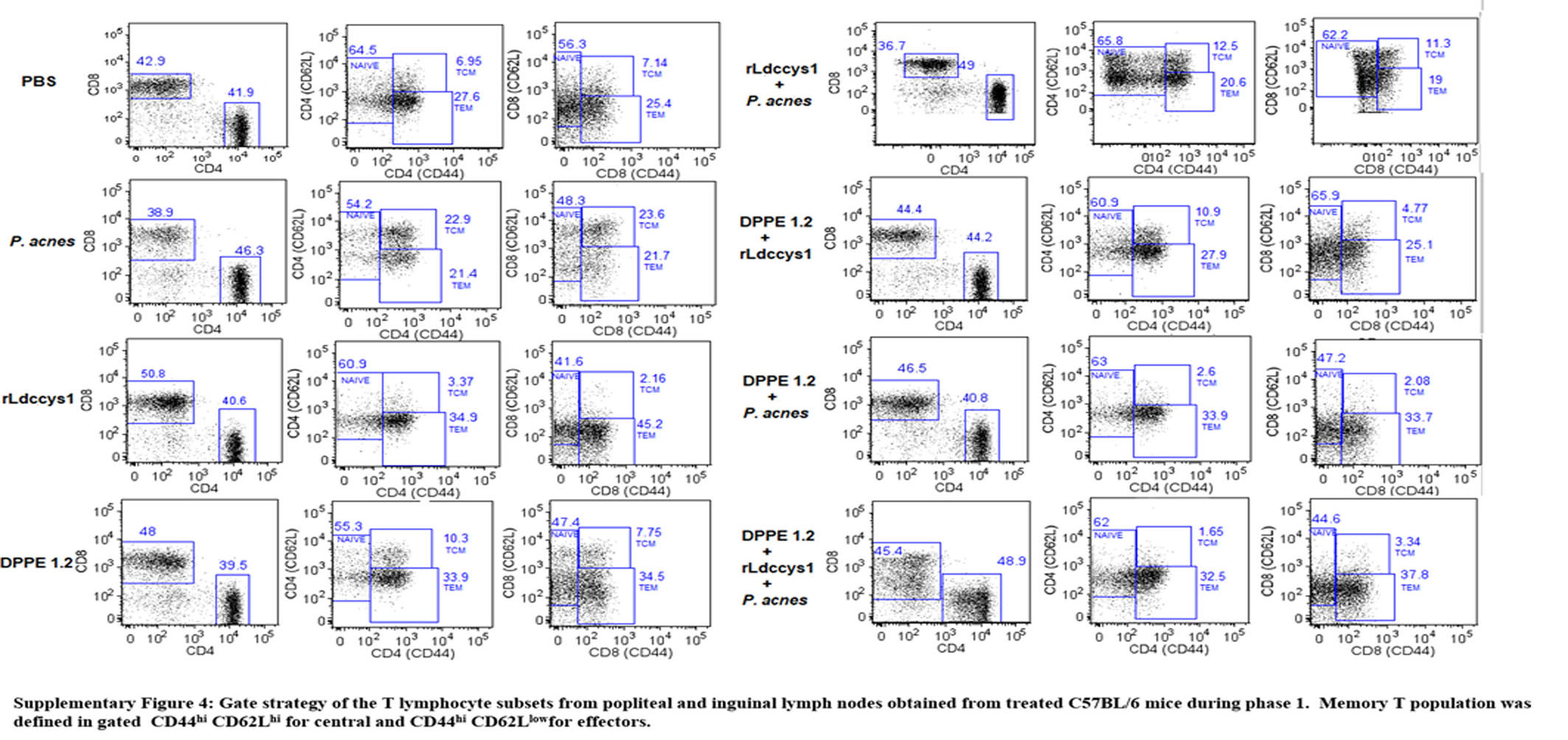

Supplement: Supplementary file 4 [file Image_4.JPEG]

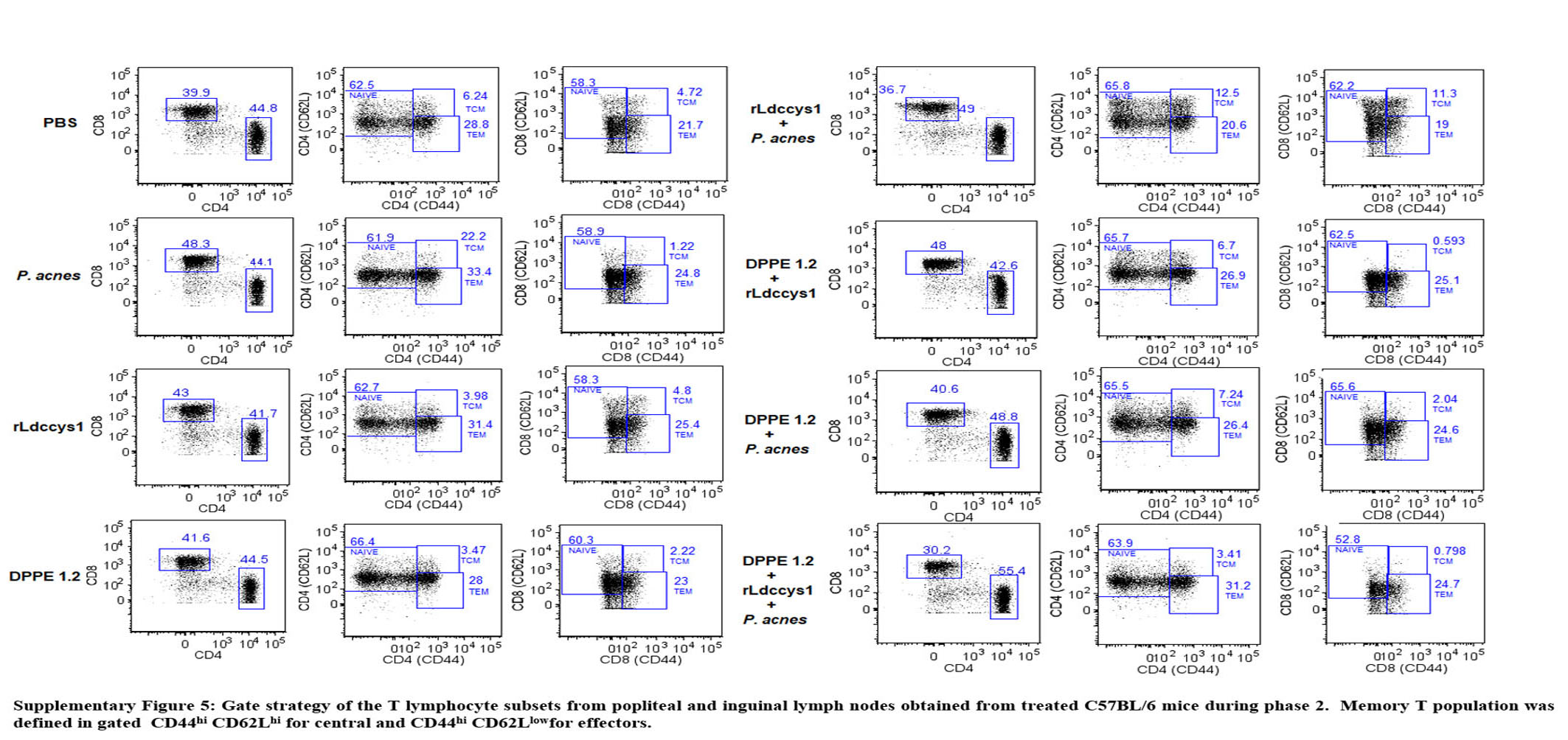

Supplement: Supplementary file 5 [file Image_5.JPEG]

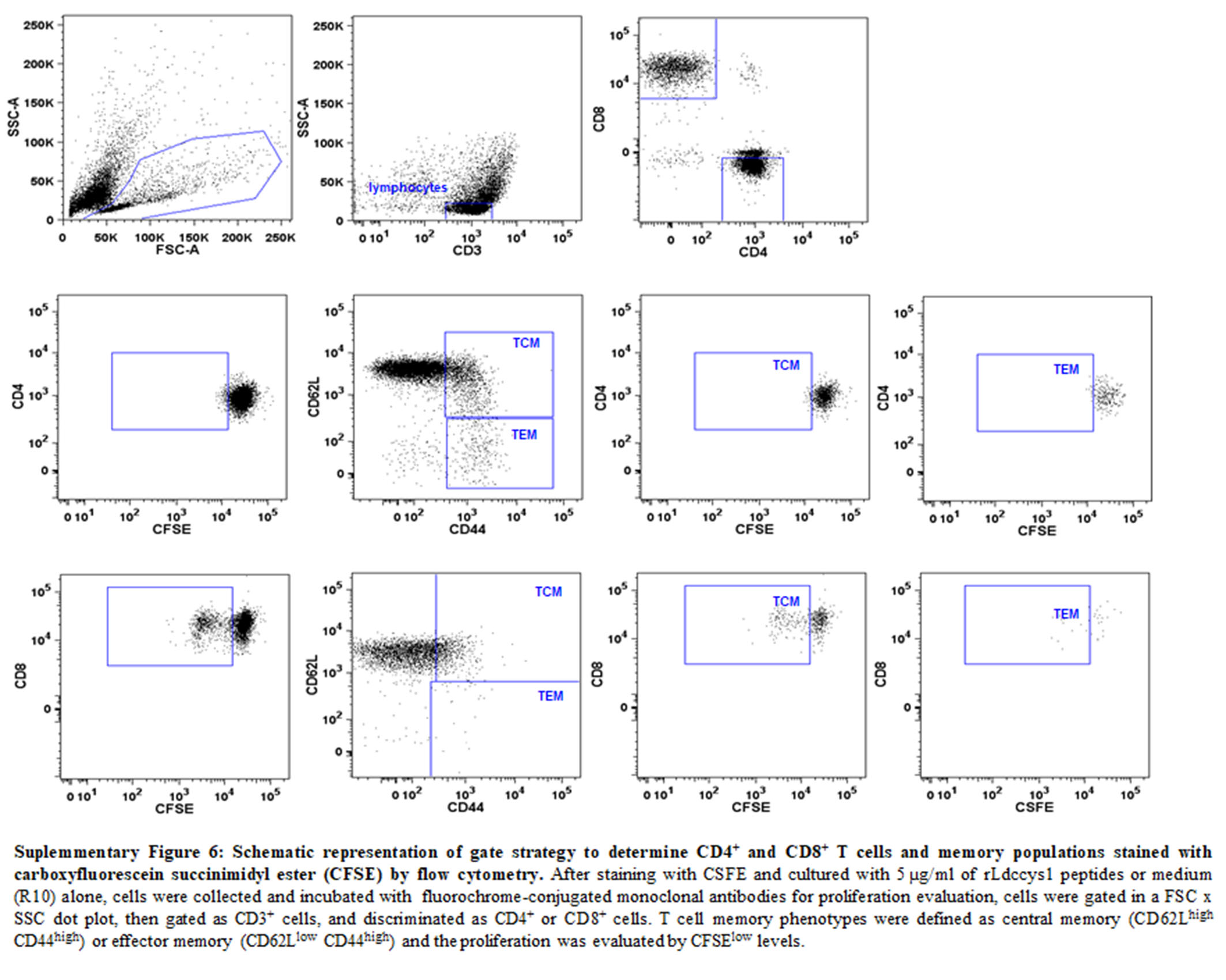

Supplement: Supplementary file 6 [file Image_6.jpg]

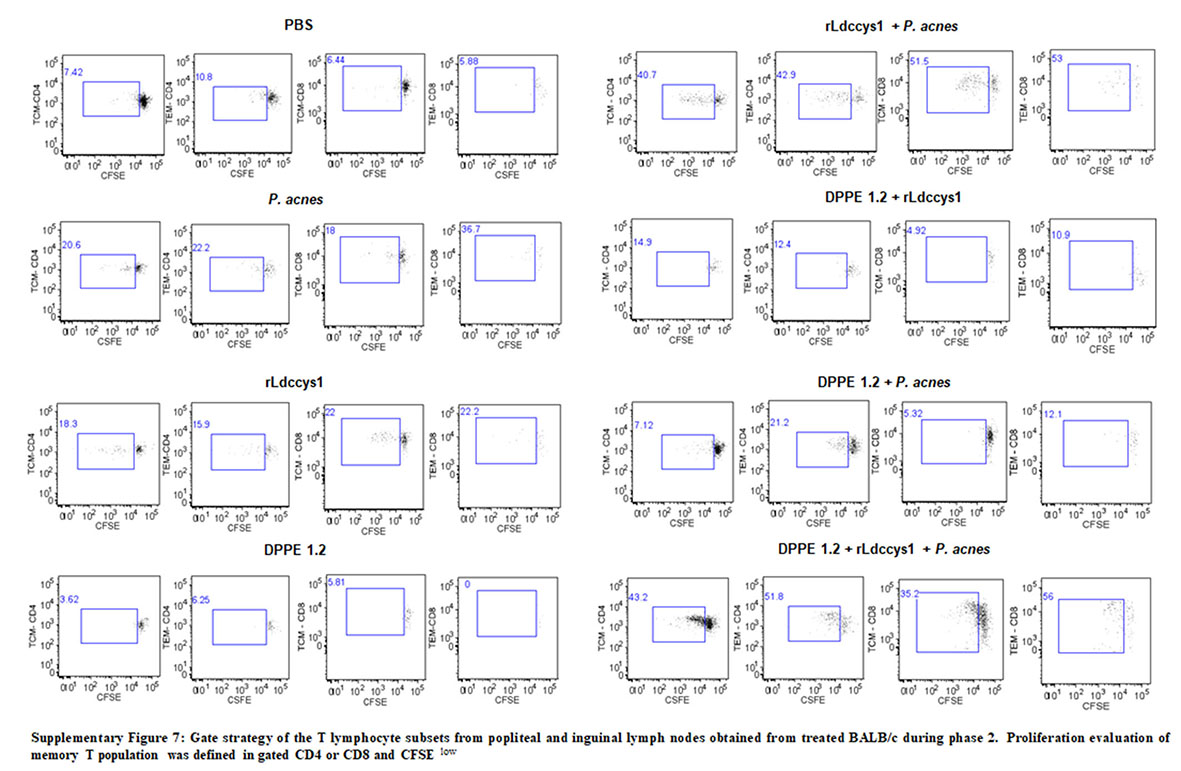

Supplement: Supplementary file 7 [file Image_7.JPEG]
